# A brief review on molecular, genetic and imaging techniques for HCV fibrosis evaluation

**DOI:** 10.1186/1743-422X-8-53

**Published:** 2011-02-08

**Authors:** Waqar Ahmad, Bushra Ijaz, Sana Gull, Sultan Asad, Saba Khaliq, Shah Jahan, Muhammad T Sarwar, Humera Kausar, Aleena Sumrin, Imran Shahid, Sajida Hassan

**Affiliations:** 1 Applied and Functional Genomics Laboratory, Centre of Excellence in Molecular Biology, University of the Punjab, Lahore, Pakistan

## Abstract

**Background:**

Chronic HCV is one of the major causes of morbidity and mortality in the present day world. The assessment of disease progression not only provides useful information for diagnosis and therapeutic supervision judgment but also for monitoring disease. Different invasive and non invasive methods are applied to diagnose the disease from initial to end stage (mild fibrosis to cirrhosis). Although, liver biopsy is still considered as gold standard to identify liver histological stages, an assessment of the disease development based on non-invasive clinical findings is also emerging and this may replace the need of biopsy in near future. This review gives brief insight on non-invasive methods currently available for predicting liver fibrosis in HCV with their current pros and cons to make easier for a clinician to choose better marker to assess liver fibrosis in HCV infected patients.

**Methods:**

More than 200 studies regarding invasive and noninvasive markers available for HCV liver disease diagnosis were thoroughly reviewed. We examined year wise results of these markers based on their sensitivity, specificity, PPV, NPV and AUROCs.

**Results:**

We found that in all non-invasive serum markers for HCV, FibroTest, Forn's Index, Fibrometer and HepaScore have high five-year predictive value but with low AUROCs (0.60~0.85) and are not comparable to liver biopsy (AUROC = 0.97). Even though from its beginning, Fibroscan is proved to be best with high AUROCs (> 0.90) in all studies, no single noninvasive marker is able to differentiate all fibrosis stages from end stage cirrhosis. Meanwhile, specific genetic markers may not only discriminate fibrotic and cirrhotic liver but also differentiate individual fibrosis stages.

**Conclusions:**

There is a need of marker which accurately determines the stage based on simplest routine laboratory test. Genetic marker in combination of imaging technique may be the better non invasive diagnostic method in future.

## 1. Introduction

Chronic Hepatitis C (HCV) is one of the major causes of liver fibrosis, with distortion of the hepatic architecture, and ultimate progression to cirrhosis. Approximately more than 3% of the total world population is chronically infected with HCV and due to gradual increase in the prevalence of HCV; future burden of chronic HCV is predicted to raise at least 3 fold by the year 2020. Common causes of liver fibrosis are viral hepatitis and steato hepatitis with alcohol or obesity. Fibrosis caused by excessive deposition of extracellular matrix (ECM) by histological and molecular reshuffling of various components like collagens, glycoproteins, proteoglycans, matrix proteins and matrix bound growth factors. These changes can lead to metabolic and synthesis impairment to hepatocytes, epithelial cells and hepatic stellate cells (HSC). HSC activation the main step leading to fibrosis, involves several changes in liver like fibrogenesis, proliferation, contractility, chemotaxis, matrix degradation and cytokine release. Fibrosis can be defined as net result of the balance between ECM production and degradation. As ECM tissues not only involve matrix production but also matrix degradation leading to ECM remodeling, fibrosis is potentially a reversible process in early stages (advance stages in some cases) [1-6].

**Figure 1:**
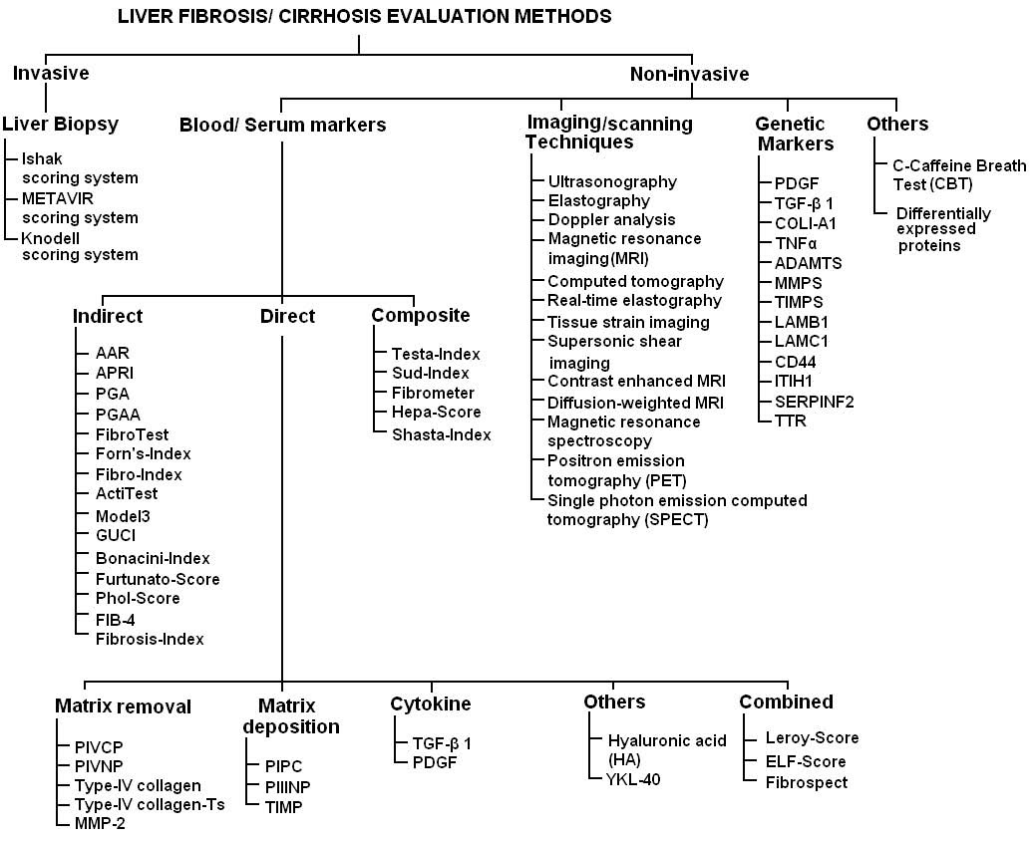
**Schematic diagram of noninvasive methods used to assess liver fibrosis and cirrhosis in HCV or co-infected patients**.

Fibrosis stages information not only indicate treatment response but also reflect/indicate cirrhosis development disaster. We can evaluate fibrosis in HCV infected patients invasively or non-invasively. Liver biopsy an invasive method is used for histological scoring and still used as reference test for fibrosis staging. With the increasing knowledge of molecular biology, genetics and availability of modern imaging techniques, many clinicians and related scientists developed several non-invasive methods to assess liver fibrosis and cirrhosis. These markers need to be more precise, reproducible and non-invasive to evaluate liver fibrosis in HCV infected patients. Therefore, an assessment of the disease development based on clinical findings is still critical for patients infected with HCV. The accuracy of a serological test either individually or in combination is given as the area under the curve (AUC) of the receiver operator characteristic (ROC) of specific serum diagnosis test. In the meantime, genetic marker should reflect differential expression in different fibrosis stages [4,7-13]. This article will focus on the technologies that can be used to assess hepatic fibrosis in HCV infected patients with unequal values. Figure 1 shows an outline of possible methods used for fibrosis evaluation in HCV infected patients.

## 2. Invasive Method

In clinical practice, grading and staging involve semi-quantitative scoring systems, and elementary lesion expressed as a numerical value [14,15]. Three scoring systems, Knodell, Ishak and Metavir are extensively used to assess fibrosis [16-18]. In Metavir system, one of the most clinically validated systems; F0-F1 is considered none to mild, F2-F3 moderate to advance fibrosis and F4 as cirrhosis. Liver biopsy, an invasive method is considered the gold standard to identify liver fibrosis. Unfortunately, procedure of liver biopsy is invasive, expensive with severe side effects leading to death and not suitable for all patients. Other limitations of liver biopsy comprises sampling error, intra and inter observer variation and somehow static, not accurately predict disease progression [19,20].

## 3. Non-invasive Methods

Non-invasive methods can be classified as serum, genetic and imaging techniques. These markers are addressed below in detail.

## 4. Serum markers

Serological markers refer to the measurement of one or more molecules within blood or serum correlating to hepatic fibrosis [21-23]. There are several proposed serological markers or combinations of serum markers for hepatic fibrosis measurement. Their levels vary by changes in their clearance, metabolism, and excretion, and their significant contribution from non-hepatic sources, such as, bones, joints, lungs, kidneys and skin [24,25]. Proposed hepatic fibrosis serological markers can be divided in three categories as direct, indirect or composite. Combinations of both direct and indirect, markers are taking place as an emerging and promising alternative to liver biopsy [26-29]. Figure 1 gives a brief idea about the non-invasive methods used for fibrosis and cirrhosis prediction in HCV infected patients.

### 4.1. Direct serum markers

Direct serum markers reflect ECM turnover, balance between hepatic fibrogenesis and fibrolysis, and in the deposition and removal of ECM. Levels of direct serum markers are elevated during disease progression and an independent association between stage of fibrosis and direct markers was observed [30-32]. Some of the markers reported are discussed below.

#### 4.1.1. Matrix deposition and removal markers

These may be classified into following

##### Procollagen I carboxy terminal peptide (PICP), Procollagen III amino-terminal peptide (PIIINP) and Type IV collagen

PICP and PIIINP released into the serum during matrix removal and deposition. PIIINP reflects the stage of fibrosis and known to be elevated in chronic liver disease. PIIINP is a good inflammatory score predictor as compared to fibrosis. PICP usually indicates cirrhosis and used for quantifying disease severity. However, it reflects alcohol etiology better than diagnosis of chronic liver disease. Type IV serum collagen reflects matrix degradation and increased in chronic liver disease. Murawaki *et al *(1996) established the cutoff value of 110 ng/mL for stages greater than F2 and 130 ng/mL for F3 fibrosis stage [33-37].

##### Matrix metalloproteinase (MMP's) and tissue inhibitor of metalloproteinases (TIMPs)

MMP's enzymes produced intracellularly and secreted in a pro-enzyme form that requires cleavage by cell surface mechanisms control matrix degradation. Although these proteins act both to degrade and deposition of ECM, also involve in activation of growth factor, effect on cell proliferation and inhibition of apoptosis; their association with liver fibrosis is not clear [4,23]. TIMPs also increased during HCV infection, while a decrease is reported after interferon therapy. These have high diagnostic ability to detect cirrhosis [38].

##### Cytokines

Two types of cytokines TGF-β 1 (transforming growth factors β 1) and PDGF (platelet derived growth factor) are mainly used to assess the fibrosis progression. TGF-β 1 is the dominant stimulus for producing extracellular matrix and it showed a significant correlation with degree of hepatic fibrosis. A significant association was found between TGF-β 1 serum levels and fibrosis progression. Serum level of PDGF has also showed high ability as serum marker for fibrosis progression [39-41].

#### 4.1.2.Combined direct markers

##### FibroSpect

FibroSpect assay is a combination of three parameters: HA, TIMP-1 and alpha-2-macroglobulin and can differentiate between no/mild and moderate/severe fibrosis [42,43]. Maximum sensitivity and specificity of this assay was observed at two extreme stages (F0 and F4). This assay was further developed by adding YKL-40 serum marker for assessing Ishak stages and digital quantification of fibrosis [23].

##### ELF

European liver fibrosis group (ELF) developed an algorithm consisted of HA, PIIINP, TIMP-1 and age. However this assay showed low performance while predicting fibrosis in chronic HCV patients [44].*Leroy Score*

This score was developed by Leroy *et al *and contains PIIINP and MMP-1 as basic components. It can differentiate between mild and significant fibrosis [45].

#### 4.1.3.Others

##### Hyaluronic acid (HA)

HA is best validated, an essential component of extracellular matrix of body tissues. HA levels increases with the fibrosis progression and correlate with the degree of fibrosis and inflammation in chronic HCV patients. The diagnostic accuracy of HA is better than that of PIIINP [32,35,46-49].

##### Chondrex, human cartilage glycoprotein (YKL-40)

In liver fibrosis, YKL-40 plays role in tissue degradation and extracellular matrix remodeling. YKL-40 level is observed to decrease after interferon therapy. In a combination of different direct serum markers, HA and YKL-40 were more useful for monitoring fibrosis progression with 80% PPV of predicting stage specific fibrosis. A significant association of HA with liver fibrosis was observed when compared with TGF-β1 [50-53].

Table 1 briefly describes a year wise overview of the AUROCs, PPV, NPV, sensitivity and specificity of direct serum markers used in various studies to predict fibrosis and cirrhosis in HCV infected patients. Direct serum markers; HA, YKL-40 and ELF were able to predict significant fibrosis as well as cirrhosis with AUROC 0.70-0.85. However, these markers showed low sensitivity and NPV for predicting fibrosis and high efficiency to detect cirrhosis.

**Table 1:** Diagnostic accuracies of direct serum markers

Markers	Study	Year	Prognosis	Sen	Spe	PPV	NVP	AUC
**ELF Score**	Rosenberg [44]	2004	Fibrosis	90	41	99	92	0.80
			Cirrhosis	-	-	-	-	0.89
	Cales [79]	2005	Fibrosis	-	-	-	-	0.88
	Parkes [12]	2006	Fibrosis	-	-	-	-	0.78
	Lee [107]	2010	Cirrhosis					0.70
**FibroSpect**	Patel [42]	2004	Fibrosis	77	73	74	75	0.83
	Cales [79]	2005	Fibrosis	-	-	-	-	0.87
	Zaman [43]	2007	Fibrosis	72	74	61	82	0.82
**HA**	Guechot [34]	1996	Fibrosis	64	91	-	-	0.86
			Cirrhosis	79	89	-	-	0.92
	Murawaki [47]	2001	Fibrosis	75	80	77	78	0.86
			Cirrhosis	50	79	42	84	0.92
	Halfon [49]	2005	Fibrosis	14	99	94	57	0.75
			Cirrhosis	31	99	57	96	0.89
	Suzuki [48]	2005	Fibrosis	85	80	51	96	0.89
			Cirrhosis	-	-	-	-	0.92
	Saitou [51]	2005	Fibrosis	80	80	80	80	0.92
	Parise [81]	2006	Fibrosis	85	71	-	-	0.88
			Cirrhosis	91	82	-	-	0.91
**Leroy Score**	Leroy [45]	2004	Fibrosis	43	64	45	40	-
**PIIINP**	Guechot [34]	1996	Fibrosis	70	63	-	-	0.69
			Cirrhosis	60	74	-	-	0.73
	Murawaki [47]	2001	Fibrosis	74	75	75	92	-
			Cirrhosis	64	59	33	84	-
	Saitou [51]	2005	Fibrosis	78	75	76	77	0.75
			Cirrhosis	77	66	69	67	0.79
**PIVNP**	Murawaki [47]	2001	Fibrosis	70	73	71	72	-
			Cirrhosis	63	73	41	87	-
**TIMP**	Murawaki [47]	2001	Fibrosis	79	56	63	73	-
			Cirrhosis	82	54	34	94	-
	Boeker [38]	2002	Fibrosis	52	88	-	-	0.71
			Cirrhosis	100	75	-	-	0.90
**YKL-40**	Saitou [51]	2005	Fibrosis	78	81	80	79	0.81
			Cirrhosis	80	71	73	78	0.80

## 5. Indirect serum fibrosis markers

The other category of serum marker is indirect markers that are based on the disturbance of hepatic function or structure.

### 5.1. Serum ALT, AST and AFP levels

Serum ALT released from liver tissue into the circulation in proportion to the degree of hepatocellular damage due to viral infections and toxic substances [54,55]. ALT is thought as one of the more sensitive marker of liver injury and disease progression [56-58]. However, ALT enzymatic activity may not always reflect the degree of hepatic damage as about 26% patients have persistently normal ALT levels but have a histological score greater than A1F1 [59]. Serum AST levels are most important predictor of histological activity than ALT [60-62]. Serum AFP is alpha-1-globulin secreted by fetal hepatocytes and fetal gastrointestinal tract. Elevated serum AFP levels are associated with acute and chronic HCV, toxic liver injury concentrations and correlate with tumor size and decrease or normalize after tumor removal. Elevated AFP levels are observed in cirrhotic patients [63-66].

### 5.2. Platelet count (PLT)

Decreased production of thrombopoietin by hepatocytes and reduced platelet production is associated with fibrosis progression. Platelet count (< 150 × 10^9^/L < 100) can differentiate cirrhotic (F4) from fibrosis (F1-F3) in 75-80% chronic HCV patients [67-70].

### 5.3. Prothrombin time (PT)

PT reflects the synthesis capacity of the liver and essential mechanism of blood coagulation. Its clinical reference range is usually around 12-15 seconds. Prolonged PT is associated with esophageal varices and is one of the earliest indicators of liver cirrhosis [71-73].

### 5.4. AST/ALT ratio (AAR)

Sheth *et al. *reported an AST/ALT ratio ≥ 1 having 100% PPV for the presence of cirrhosis in chronic HCV patients [74]. Reedy *et al. *observed that AAR failed to predict cirrhosis accurately in HCV patients [75], while Giannini *et al. *reported high diagnostic accuracy of the AAR for prediction of cirrhosis in HCV infected patients [76]. However, many authors could not able to find high accuracy of this marker [4,70,77].

### 5.5. AST to platelet ratio Index (APRI)

APRI was the simplest and accurate test for significant liver fibrosis and cirrhosis [28]. Several authors verified this marker for fibrosis and cirrhosis and found it better than AAR. However, APRI was unable to identify individual stages of fibrosis [77-86].

### 5.6. PGA and PGAA Index

PGA was known to be the original index of hepatic fibrosis in 1990 s and combines gamma glutamyl transferase (γGT), apolipoprotein A1 (PGA) and prothrombin index. PGAA index is modified form of PGA index by the addition of alpha-2-macroglobulin, resulted in its improved version. The diagnostic accuracy of the PGA and PGAA for detecting cirrhosis reported between 66-72% and 80%, respectively [87-92].

### 5.7. FibroTest/FibroSure

FibroTest is the combination of five markers: alpha-2-macroglobulin, haptoglobin, apolipoprotein A_1_, GGT and total bilirubin [26,80]. This marker has 75% sensitivity and 85% specificity with reproducibility for fibrosis diagnosis [83-85]. However, Rossi *et al. *reported low AUROC (0.739) for significant fibrosis with NPV and PPV 85% and 78%, respectively. Meanwhile, FibroTest is validated and suggested as an alternative to liver biopsy in chronic HCV patients [93-105].

### 5.8. Fibro Index

It combines three markers; AST, platelet count and gamma globulin. AUROC for prediction of significant fibrosis was 0.83 [106].

### 5.9. Forns Index

This index is based on four available variables; age, GGT, platelet count and cholesterol levels in a study on HCV patients, included both test and validation cohorts [27]. The limitation of this index was the identification of advance liver disease with minimal fibrosis [79,80,106,107].

### 5.10. ActiTest

ActiTest reflects both necroinflamatory activity and liver cirrhosis. It is modified form of Fibrotest with addition of ALT level (26). Fibrotest and ActiTest were found to be potential non-invasive assays for the assessment of hepatic fibrosis and necro-inflammatory activity in pediatric patients with chronic HCV in comparison with liver biopsy [90,91,108].

### 5.11. SteatoTest

It incorporates the FibroTest, ALT, body mass index, serum cholesterol, triglycerides and glucose adjusted for age and gender. It has 63% PPV for steatosis prevalence with 93% NPV [109].

### 5.12. Model 3

This model is based on AST, platelet count and prothrombin time expressed as international normalized ration (INR). Patients with liver cirrhosis can be excluded at cutoff value of < 0.20 with 99% NPV [110,111].

### 5.13. Goteborg University Cirrhosis Index (GUCI)

Islam *et al. *found strong association between AST, prothrombin-INR and platelet count. By using a cutoff value 1.0, the sensitivity and specificity for the diagnosis of cirrhosis was 80% and 78% respectively, while the NPV and PPV were 97% and 31%, respectively [112].

### 5.14. Fibrosis Index

This index comprises of platelet count and albumin contents. It can differentiate significant fibrosis and cirrhosis from mild fibrosis [113].

### 5.15. Phol Score

This index comprises of AST, ALT and platelet count. It showed great accuracy for discriminating significant fibrosis and cirrhosis with high PPV and NPV. However, it showed limited ability to predict fibrosis in later study [114,115].

### 5.16. Bonacini Index

This index incorporates ALT/AST ratio, INR and platelet count. It showed 94% specificity for predicting significant fibrosis in initial cohort [116].

Table 2 represents the diagnostic accuracies of indirect serum markers. Indirect serum markers are easily available and routinely used. These markers have the ability to differentiate fibrosis and cirrhosis but lesser extent to direct serum markers. APRI and FibroTest are most validated serum markers with AUROC range between 0.60-0.85 for predicting fibrosis and cirrhosis.

**Table 2:** Diagnostic accuracies of indirect serum markers

Markers	Study	Year	Prognosis	Sen	Spe	PPV	NVP	AUC
**AAR**	Sheth [74]	1998	Cirrhosis	53	100	100	81	0.85
	Afdhal [4]	2004	Fibrosis	47	-	-	88	-
			Cirrhosis	-	96	74	-	-
	Lackner [70]	2005	Fibrosis	53	100	-	-	0.57
			Cirrhosis	36	90	41	87	0.73
	Fuji [77]	2009	Fibrosis	-	-	-	-	0.56
**ActiTest**	Imbert-Bismut [26]	2001	Fibrosis	91	42	-	-	0.79
	Halfon [100]	2008	Fibrosis	90	38	-	-	0.75
**APRI**	Wai [28]	2003	Fibrosis	41	95	64	90	0.88
			Cirrhosis	-	-	57	-	0.94
	Cales [79]	2005	Fibrosis	-	-	-	-	0.79
	Bourliere [80]	2006	Fibrosis	22	95	63	76	0.71
			Cirrhosis	38	96	96	40	0.81
	Parise [81]	2006	Fibrosis	85	66	-	-	0.82
			Cirrhosis	73	81	-	-	0.84
	De Ledinghen [82]	2006	Cirrhosis	-	-	-	-	0.73
	Halfon [83]	2007	Fibrosis	77	66	61	80	0.76
			Cirrhosis	100	83	18	100	0.92
	Leroy [84]	2008	Fibrosis	39	95	88	62	0.79
	Cales [85]	2008	Fibrosis	62	83	80	67	0.78
			Cirrhosis	-	-	-	-	0.84
	Kamphues [86]	2010	Fibrosis	70	63	80	80	0.68
			Cirrhosis	89	44	14	97	0.63
	Fuji [77]	2009	Cirrhosis	-	-	-	-	0.76
**Fibro Index**	Koda [106]	2007	Fibrosis	36	97	94	59	0.83
**Fibrosis Index**	Ohta [113]	2006	Fibrosis	68	71	75	81	0.85
**FibroTest**	Imbert-Bismut [26]	2001	Fibrosis	87	59	63	85	0.87
			Cirrhosis					
	Bedosa [102]	2003	Fibrosis	27	97	90	55	-
	Myers [101]	2003	Fibrosis	-	95	88	-	0.83
	Poynard [90]	2003	Fibrosis	-	-	-	-	0.73
	Rossi [97]	2003	Fibrosis	83	52	52	83	0.74
	Colletta [103]	2005	Fibrosis	64	31	33	62	-
	Bourliere [80]	2006	Fibrosis	55	90	73	79	0.82
	De Ledinghen [82]	2006	Cirrhosis	-	-	-	-	0.73
	Halfon [83]	2007	Fibrosis	67	80	70	78	0.79
			Cirrhosis	85	74	11	99	0.86
	Leroy [84]	2008	Fibrosis	57	85	78	68	0.80
	Cales [85]	2008	Fibrosis	67	82	80	70	0.81
	Shaheen [104]	2008	Fibrosis	47	90	-	-	0.81
			Cirrhosis	-	-	-	-	0.90
	Cales [105]	2010	Fibrosis	-	-	-	-	0.81
			Cirrhosis	-	-	-	-	0.88
**Forn's Index**	Forn [27]	2002	Fibrosis	94	51	40	96	0.78
	Cales [79]	2005	Fibrosis	-	-	-	-	0.82
	Bourliere [80]	2006	Fibrosis	30	96	65	83	0.76
	Koda [106]	2007	Fibrosis	-	-	-	-	0.79
**Model 3**	Lok [110]	2005	Cirrhosis	10	100	100	86	0.78
**PGA**	Teare [87]	1993	Fibrosis	94	81	-	-	-
			Cirrhosis	-	-	86	-	-
	Poynard [90]	2003	Fibrosis	91	81	-	-	-
	Poynard [91]	2004	Fibrosis	79	89	-	-	-
**PGAA**	Naveau [92]	2005	Cirrhosis	89	79	-	-	0.93
**Phol Score**	Pohl [114]	2001	Fibrosis	41	99	93	85	-
	Cheung [115]	2008	Fibrosis	-	-	-	-	0.53

## 6. Composite fibrosis markers

### 6.1. FibroMeter

FibroMeter can differentiate fibrosis progression in viral disease consist of combination of HA, AST, platelet count, prothrombin index, alpha-2-macroglobulin, urea and age of the patients [105].

### 6.2. Hepascore

Hepascore is a model consisting of bilirubin, GGT, HA, alpha-2-macroglobulin, gender and age. AUROC of this test is 0.85, 0.96 and 0.94 for significant fibrosis, advanced fibrosis and cirrhosis, respectively [117-120].

### 6.3. Shasta Index

It combines HA, AST and albumin. Optimal results of this assay are observed in extreme conditions. This assay showed similar accuracy with FibroTest [121].

### 6.4. Apricot (FIB-4)

This assay combines four markers: AST, ALT, platelet count and age. This index can predict significant fibrosis in patients infected with HIV/HCV [122]. Later studies validated this index not only in co-infected patients but also in HCV infected patients [85,123,124].

### 6.5. Sud Index

This assay is also known as FPI comprises of age, AST, cholesterol, insulin resistance and alcohol intake. This index showed high specificity and PPV for detecting advance fibrosis [125].

### 6.6. Testa Index

This index relate platelet count and spleen diameter. This ratio showed 78% concordance with the histological score [126].

### 6.7. Fortunato score

This model contains fibronectin, prothrombin time, PCHE, ALT, Mn-SOD and β-NAG as essential components. It has ability to classify cirrhotic from chronic patients with high accuracy in initial and validation cohort [127].

Table 3 gives an idea about the prediction levels of combined serum markers. These markers showed high AUROCs (0.80-0.90) for predicting fibrosis and cirrhosis in HCV infected patients. FIB-4, Fibrometer and Hepascore are most precise and validated serum markers. Combined serum markers are easily available and most preferable non invasive serum markers now a day.

**Table 3:** Prognosis accuracies of combined serum markers

Markers	Study	Year	Prognosis	Sen	Spe	PPV	NVP	AUC
**FIB-4**	Sterling [122]	2006	Fibrosis	70	74	42	71	0.80
			Cirrhosis					
	De Ledingh [82]	2006	Cirrhosis	-	-	-	-	0.73
	Vallet-Pichard [123]	2007	Fibrosis	74	80	82	95	0.85
	Cales [85]	2008	Fibrosis	74	72	74	71	0.80
			Cirrhosis	-	-	-	-	0.87
	Mallet [124]	2009	Fibrosis	71	73	52	86	0.81
			Cirrhosis	-	-	-	-	0.87
	Lee [107]	2010	Cirrhosis	-	-	-	-	0.71
**Fibrometer**	Halfon [83]	2007	Fibrosis	92	87	21	100	0.94
			Cirrhosis	62	87	21	100	0.94
	Cales [85]	2008	Fibrosis	-	-	-	-	0.90
			Cirrhosis	-	-	-	-	0.90
	Cales [105]	2010	Fibrosis	-	-	-	-	0.88
			Cirrhosis	-	-	-	-	0.88
**Fortunato Score**	Fortunato [127]	2001	Fibrosis	-	94	-	-	-
**HepaScore**	Adams [117]	2005	Fibrosis	63	89	88	90	0.82
			Cirrhosis	71	89	-	-	0.90
	Bourliere [80]	2006	Fibrosis	-	-	-	-	0.82
			Cirrhosis	-	-	-	-	0.90
	Halfon [83]	2007	Fibrosis	77	63	59	80	0.76
			Cirrhosis	92	72	11	100	0.89
	Leroy [118]	2007	Fibrosis	54	84	78	64	0.79
	Leroy [84]	2008	Fibrosis	63	80	75	70	0.78
	Cales [85]	2008	Fibrosis	66	79	77	68	0.78
			Cirrhosis	-	-	-	-	0.90
	Becker [119]	2009	Fibrosis	82	65	70	78	0.81
			Cirrhosis	-	-	-	-	0.88
	Cales [105]	2010	Fibrosis	-	-	-	-	0.78
			Cirrhosis	-	-	-	-	0.89
	Guechot [120]	2010	Fibrosis	77	70	71	77	0.81
			Cirrhosis	86	74	37	97	0.88
**Shasta Index**	Kelleher [121]	2005	Fibrosis	88	72	55	94	0.87
**Sud Index**	Sud [125]	2004	Fibrosis	42	98	97	54	0.84
**Testa Index**	Testa [126]	2006	Fibrosis	78	79	-	-	0.80

## 7. Imaging/scanning techniques

Imaging techniques are rational noninvasive approach to assess liver fibrosis. Imaging techniques are not only capable to detect changes in the hepatic parenchyma, these can differentiate between moderate and severe fibrosis. However, high cost and lack of validation of concerning studies remains controversial. Brief detail of these techniques is given under, while there limitations are addressed in Table 4.

**Table 4:** Summarized imaging techniques with their limitations

Method	Technique	Limitations
**Ultrasonography**	Identification of portal hypertension	Limited capability to measure mild or moderate fibrosis and cirrhosis, contradictory results
**Elastography**	Liver stiffness	Vulnerable measurements due to narrow intercostals spaces, ascites or obesity
**Doppler Analysis**	Measures velocity of blood flow, hemodynamic variations	Limited data, lack of reproducibility, contradictory results
**Magnetic Resonance Imaging**	Observe changes in hepatic parenchyma	High cost, lack of research support
**Computed Tomography**	Identifies micro vascular permeability changes	Recent technique, not much literature is available, can not performed in renal failure and contrast agent allergic patients

### 7.1. Ultrasonography (US)

Ultrasonography detects changes appear in liver echogenicity, nodularity and signs of portal hypertension. A number of studies proposed the role of ultrasonography as a non-invasive diagnostic marker of liver fibrosis and revealed a great sensitivity of ultrasonography to detect late stages of progressive hepatic fibrosis, but a limited capability to measure mild or moderate fibrosis. Ultrasound can identify cirrhosis in patients with sensitivity of 84% and specificity of 100% and diagnose accurately 94%. Shen *et al. *observed that the echo pattern of the hepatic surface contributed to diagnostic accuracy, which was also confirmed in a separate study. However, Oberti found ultrasonography as weak diagnostic marker when compared it with other clinical and biochemical examinations [128-133].

### 7.2. Transient Elastography (FibroScan): an applicable alternative to liver biopsy

Transient elastography measures tissue stiffness. It can measure liver sample size 100 times greater than a standard biopsy sample size, as liver biopsy size strongly effects the grading of chronic viral hepatitis [134-137]. FibroScan results reported 100% sensitivity and specificity for PPV & NPV respectively (103). In a study of 935 patients Fibroscan was found to be 97% successful in grading chronic HCV infection [138]. In another study on 711 patients, liver stiffness measurements (LSM) were also found closely related to fibrosis stage [139]. Vizzutti *et al. *has also reported a good correlation between liver stiffness measurement and HVPG (hepatic venous pressure gradient) in cirrhotic patients. Success rate depends on patient body mass index, observer expertise and inter-coastal spaces with 5% failure chances. Several authors assess the performance of elastography and configure it best for the diagnosis of fibrosis [13,86,103,140-149]. A combination of FibroScan with FibroTest also gives a better understanding to detect fibrosis and cirrhosis with high AUC [104]. Table 5 briefly describes the diagnostic accuracy of FibroScan with or without combination with FibroTest. In all studies, FibroScan showed highest AUROC (> 0.90) but not more than liver biopsy (AUROC > 0.970).

**Table 5:** Diagnostic accuracy of Fibroscan with and without FibroTest

Markers	Study	Year	Prognosis	Sen	Spe	PPV	NVP	AUC
**Fibro Scan**	Ziol [13]	2005	Fibrosis	56	91	88	56	0.79
			Cirrhosis	86	96	78	97	0.97
	Colletta [103]	2005	Fibrosis	100	100	100	100	1.00
	Foucher [139]	2006	Fibrosis	64	85	90	52	0.80
			Cirrhosis	77	97	91	92	0.96
	Corpechot [145]	2006	Fibrosis	-	-	-	-	0.95
								
	Ganne-Carrie [146]	2006	Cirrhosis	79	95	74	96	0.95
	Kettaneh [138]	2007	Fibrosis	-	-	-	-	0.79
			Cirrhosis	-	-	-	-	0.91
	Shaheen [147]	2007	Fibrosis	64	87	-	-	0.83
			Cirrhosis	-	-	-	-	0.95
	Friedrich-Rust [148]	2009	Fibrosis	-	-	-	-	0.84
			Cirrhosis	-	-	-	-	0.94
	Kamphues [86]	2010	Fibrosis	72	83	96	58	0.81
			Cirrhosis	100	65	23	100	0.87
	Sanchez-Conde [149]	2010	Fibrosis	76	75	70	81	0.93
			Cirrhosis	100	94	57	100	0.99
**Fibro Scan + FibroTest**	Castera [160]	2005	Fibrosis	-	-	-	-	0.88
			Cirrhosis	-	-	-	-	0.95
	Shaheen [104]	2008	Fibrosis	47	90	-	-	-

### 7.3. Doppler analysis

Doppler measures the velocity of blood flow hemodynamic variations in hepatic vasculature, as sever fibrosis causes irregularities and abnormalities in hepatic blood vessels. Recent data indicate a close correlation between arterioportal ratio and degree of fibrosis, higher ratio indicates severe fibrosis (F3-F4) and low ratio shows moderate fibrosis (F1-F2) [150-153].

### 7.4. Magnetic resonance imaging (MRI)

MRI observes changes in hepatic parenchyma. Non-invasive prognosis of liver cirrhosis is proposed by using double contrast material-enhanced MR imaging. This can detect cirrhosis with great sensitivity and specificity of 90%. Combining Doppler ultrasonography with MRI can give a good picture of liver fibrosis in patients suffering with chronic HCV [154-156].

### 7.5. Computed tomography (CT)

CT identifies microvascular permeability changes in a model of liver fibrosis. In a latest study, the severity of liver fibrosis was predicted by heterogeneous enhancement of the liver; hepatic parameters. Perfusion calculated with a dynamic contrast-enhanced single-section CT, linked with the severity of chronic liver disease. However, no well characterized study has specifically evaluated the worth of CT in diagnosing degree of fibrosis. Therefore, currently its role in diagnosis of liver fibrosis is still lacking [157-159].

### 7.6. Fibroscan + Fibrotest

The combination of two useful noninvasive methods, fibroscan and fibrotest showed high AUROC for predicting cirrhosis [104,160].

### 7.7. Modified imaging techniques

Imaging techniques with modification like Real-time elastography, Tissue strain imaging, Supersonic shear imaging, Contrast enhanced MRI, Diffusion-weighted MRI, Magnetic resonance spectroscopy, Positron emission tomography (PET), Single photon emission computed tomography (SPECT) are also in use to evaluate liver fibrosis and cirrhosis with considerable limitations like, lack of data and expertise, high cost, radiation exposure and short half-life of the tracer in PET and SPECT.

## 8. Genetic markers for liver fibrosis evaluation

ECM metabolism is very dynamic process and required an intricate balance between ECM deposition and removal. Several genetic polymorphisms influenced by factors/cytokines and affect fibrosis progression [98]. Genome-wide analysis of abnormal gene expression showed transcript deregulations during HCC development with identification of novel serum markers differentiating between normal, mild and severe fibrosis. Advantage of genetic markers over liver biopsy is intrinsic and long life while liver biopsy represents only one time point [161-163].

Huang and colleagues developed an assay known as cirrhosis risk score (CRS), a set of seven marker genes to predict cirrhosis risk in HCV infected patients. Of the seven genes, AZIN1 and TLR4 have an identified role in hepatic fibrosis, while the identification of functional mechanism of the other 5 genes is under process. The authors suggested that fibrosis risk can be identified by host genetic factors like single nucleotide polymorphism (SNP's) [164,165].

A strong association between CXCR3-associated chemokines CXCL9 and CXCL10 with liver fibrosis suggested that they may have promise as new non-invasive markers of liver fibrosis in HCV infected patients [166,167].

CTGF expression is significantly correlated with fibrosis stages and remarkably increased in advanced stages in HCV patients. The AUROC of CTGF to discriminate between mild and advanced fibrosis is 0.842 for HCV infected patients [168].

Sharma *et al. *reported the significant association and elevated interleukin-18 (IL-18) levels in fibrotic and cirrhotic liver stages, severity of disease and necrosis in HCV patients [169].

A recent study by Caillot *et al. *used microarray technique and found a significant association of ITIH1, SERPINF2 and TTR genes expression and their related proteins with all fibrosis stages. Expression of these genes and related proteins gradually decreased during the fibrosis development to its end stage cirrhosis [170].

**Figure 2:**
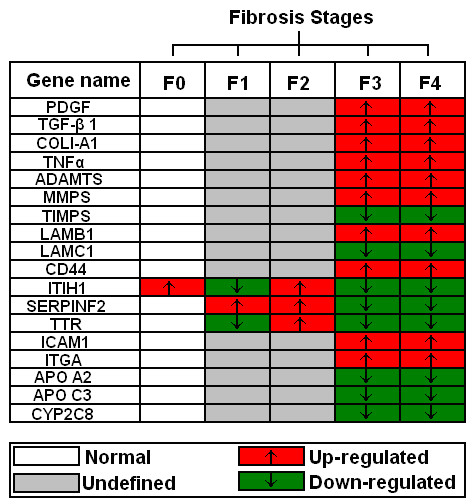
**Genes expressed differentially between different fibrosis stages (F0-F3) including cirrhosis (F4)**.

A review by Gutierrez-Reyes *et al. *briefly described role and selection of appropriate genes for fibrosis indication. They briefly explain the role of various genes like PDGF, TGF-β1, collagens COL1-A1, TNFα, interlukin, ADAMTS, MMPs, TIMPs, LAMB1, LAMC1, Cadherin, CD44, ICAM1, ITGA, APO and CYP2C8 [171]. Figure 2 represents gene clustering according to fibrosis progression on available data.

## 9. Others markers for liver fibrosis evaluation

### 9.1. C-Caffeine Breath Test (CBT)

Caffeine has high oral bioavailability and undergoes hepatic metabolism and can be use as quantitative test for liver function [172]. Park *et al. *performed caffeine breath test (CBT) and observed the correlation of orally administrated caffeine with plasma caffeine clearance and degree of liver dysfunction. Chronic patients showed significantly reduced CBT values when compared with controls [173].

### 9.2.Differentially expressed proteins

Differentially expressed proteins were identified by mass spectroscopy among different degrees of fibrosis (F0-F4) and between early (F0-F1) and late (F2-F4) fibrosis. Mac-2-binding protein, alpha-2-macroglobulin and hemopexin levels were found increased while A-1-antitrypsin, leucine-rich alpha-2-glycoprotein and fetuin-A were decreased in advanced fibrosis F4 as compared to early fibrosis F0/F1 [115].

## 10. Clinical utilization and future of non-invasive markers

Non-invasive markers should be able to differentiate between different fibrosis stages but also reflect the treatment outcome. Even though the invasive liver biopsies considered as gold standard for final assessment of liver fibrosis, non-invasive markers are risk free, reflect the liver status and may offer an attractive alternative to liver biopsy in future. However, none of currently available serum markers completely fulfill these criteria. The outcome of non-invasive markers in several studies is not same. Due to this, non-invasive markers are used in parallel to liver biopsy and not in position to completely replace liver biopsy till date.

Poynard *et al. *reported the effect of interferon plus ribavirin before and after therapy with respect to FibroTest and Actitest scores. A substantial reduction in FibroTest and Actitest was observed in patients who had showed a sustained virological response [81,90,115]. Several other studies reported the down level of serum markers like HA, YKL-40, TIMP-1 and PIIINP after interferon therapy. In these studies, level of serum markers continue to fall following treatment but most often return to permanent levels with biochemical and virological relapse. These findings suggest that these assays may be useful for initial staging of disease progression as well as histological response to therapy [174-177]. Fibroscan showed positive correlation with fibrosis stages. However, it is reported that AUROC value of Fibroscan and FibroTest must be improved as their values fall in treated patients irrespective of their virological response [178,179]. Furthermore, HCV clearance is associated with a significant reduction in non-invasive fibrosis serological markers like FibroTest, Forns Index, age-platelet ratio index, Shasta, FIB-4, Hepascore and FibroMeter [180]. Patel *et al. *compared two commercially available serum marker panels Fibrosure and Fibrospect-II in HCV patients during interferon-based therapy. Both assays showed comparable performance for differentiating mild fibrosis from moderate-severe stage [181]. Imaging techniques also have some technical limitations. These are very expensive and are not easy to handle. Their presence in each hospital or laboratory is not possible especially in poor countries. On the other hand genetic markers showed a great variability for detecting cirrhosis and fibrosis. They are also able to differentiate among fibrosis stages. But a lot of work is needed for them to become an integral part of hepatic analysis.

## 11. Conclusions

Our study showed that there are only three to four markers or set of marker that are used continuously based on their precision and accuracy in various studies for fibrosis and cirrhosis prediction. In serum non-invasive markers, FibroTest, Forn's Index, Fibrometer and HeapaScore have a high five-year prognostic value but not compared to liver biopsy (AUROC = 0.97), while Fibroscan showed maximum accuracy nearer to liver biopsy (AUROC > 0.90). Recently, genetic markers showed differential gene expression in different fibrosis stages, but these are not frequently available in all labs. Imaging techniques like ultrasound and elastography not only used to diagnose liver fibrosis but also monitor disease progression. However, genetic markers showed high ability to distinguish not only mild and advance stages of liver fibrosis but also differentiate between intermediate fibrosis stages. Although present published literature do not provide any evidence for non-invasive markers to become an integrated part of the complete assessment of liver fibrosis in HCV patients, a combination of two or more serum markers with imaging techniques may improve the accuracy of diagnosis.

## Competing interests

The authors declare that they have no competing interests.

## Authors' contributions

AW, IB, GS, AS and HS designed the study, analyze the data and wrote paper. JS, KS, SMT, KH, SA and SS checked the revised manuscript thoroughly and confirmed all the data given in manuscript. All work was performed under supervision of HS. All authors read and approved the final manuscript.

## Authors' information

Shah Jahan, Saba Khaliq and Samrin A (PhD in Molecular biology), Bushra Ijaz (M Phil Molecular Biology), Waqar Ahmad (M Phil Chemistry) and Gull S (MSc Biochemistry) are Research Officer; Sawar MT and Shahid I are Phd scholars, Asad S is MPhil scholars, while Sajida Hassan (PhD Molecular Biology) is Principal Investigator at CEMB, University of the Punjab, Lahore
